# Modulation of neuronal dynamics by sustained and activity-dependent continuous-wave near-infrared laser stimulation

**DOI:** 10.1117/1.NPh.11.2.024308

**Published:** 2024-05-17

**Authors:** Alicia Garrido-Peña, Pablo Sanchez-Martin, Manuel Reyes-Sanchez, Rafael Levi, Francisco B. Rodriguez, Javier Castilla, Jesus Tornero, Pablo Varona

**Affiliations:** a Grupo de Neurocomputación Biológica, Departamento de Ingeniería Informática, Escuela Politécnica Superior, Universidad Autónoma de Madrid, Madrid, Spain; b Hospital los Madroños, Center for Clinical Neuroscience, Brunete, Spain

**Keywords:** continuous-wave NIR laser neurotechnology, neuronal excitability, activity-dependent optical stimulation, ionic channel dynamics, computational models, open-source techniques

## Abstract

**Significance:**

Near-infrared laser illumination is a non-invasive alternative/complement to classical stimulation methods in neuroscience but the mechanisms underlying its action on neuronal dynamics remain unclear. Most studies deal with high-frequency pulsed protocols and stationary characterizations disregarding the dynamic modulatory effect of sustained and activity-dependent stimulation. The understanding of such modulation and its widespread dissemination can help to develop specific interventions for research applications and treatments for neural disorders.

**Aim:**

We quantified the effect of continuous-wave near-infrared (CW-NIR) laser illumination on single neuron dynamics using sustained stimulation and an open-source activity-dependent protocol to identify the biophysical mechanisms underlying this modulation and its time course.

**Approach:**

We characterized the effect by simultaneously performing long intracellular recordings of membrane potential while delivering sustained and closed-loop CW-NIR laser stimulation. We used waveform metrics and conductance-based models to assess the role of specific biophysical candidates on the modulation.

**Results:**

We show that CW-NIR sustained illumination asymmetrically accelerates action potential dynamics and the spiking rate on single neurons, while closed-loop stimulation unveils its action at different phases of the neuron dynamics. Our model study points out the action of CW-NIR on specific ionic-channels and the key role of temperature on channel properties to explain the modulatory effect.

**Conclusions:**

Both sustained and activity-dependent CW-NIR stimulation effectively modulate neuronal dynamics by a combination of biophysical mechanisms. Our open-source protocols can help to disseminate this non-invasive optical stimulation in novel research and clinical applications.

## 1 Introduction

Effective neural stimulation is an essential tool to study brain dynamics. Many techniques have risen since the first use of electrical, chemical, and mechanical stimulation, e.g., see Refs. 1–4. Optical methods are also widely spread, as they allow visualization^5^ and stimulation in a less invasive manner. One example is optogenetics,^6^^–^^9^ which is effective in modifying neural activity with high spatio-temporal resolution. Another example of non-invasive stimulation is transcranial magnetic stimulation,^10^ which is succeeding in clinical applications. However, they both present limitations such as the need to genetically modify the living system or restricted spatial precision, respectively. In this context, infrared laser stimulation is an optical technique that has risen in popularity in the last decade. From its first applications,^11^^,^^12^ studies have shown its ability for modulating action potentials (APs) in different systems.^13^^–^^19^ Beyond its potential as a research stimulation technique, it has also been tested for clinical use, e.g., in Parkinson’s disease, reversing brain age-related effects or depression treatment.^20^^–^^24^ This neural stimulation method is so attractive because of the wide range of possibilities that can provide for non-invasive neuromodulation offering high temporal and spatial precision.

The identification of the biophysical source of infrared neuromodulation is still under discussion as it has strong implications for applications in multiple contexts. It is difficult to associate this modulation to a single specific cause, since neural systems have distinct biophysical components reactive to the irradiation. However, most of the results point to a photo-thermal effect where the excitation driven by the laser stimulation might be caused by temperature gradient.^25^ In addition, different candidates to explain the change in neural activity have been suggested, such as capacitance,^17^^,^^26^ specific modulation of channels sensitive to temperature as TRPV4,^27^ acceleration of ionic channels,^13^ or altering the ${\mathrm{Ca}}^{2+}$ cycle possibly mediated by modulation of mitochondrial activity.^23^^,^^28^^,^^29^

Distinct types of infrared laser and action modes, in terms of the power, duration, frequency of stimulation, and wavelength have been used in previous studies, see Refs. 11, 12, and 30. The effect is highly dependent on the stimulation configuration. Most works have focused on pulsed lasers to induce spiking activity due to their stronger temperature gradient production. However, some clinical studies have successfully applied continuous-wave (CW) laser for brain stimulation.^23^

The use of closed-loop techniques has a large potential in neuroscience, for both physiological and clinical research studies,^2^^,^^31^^–^^37^ since they allow adjusting the stimulation to the context of the ongoing neural dynamics and the specific condition of the targeted system/subject. Some of these tools have been developed with open-source approaches, including optical techniques, e.g. Refs. 38–42, promoting the accessibility, reproducibility, and standardization of the studies and methods. However, near-infrared (NIR) lasers have been used with fixed/periodic stimulus and, to the best of our knowledge, they have not been exploited in activity-dependent protocols.

Here, we explore the effect of continuous-wave near-infrared (CW-NIR) laser on the dynamics of individual neurons in sustained and activity-dependent stimulation protocols. We employ a laser with constant optical power density on the sample for these two modalities. In the first case, the laser stimulation is sustained—the duration of the illumination is constant for more than 1 min—and in the second case, it is driven in an activity-dependent manner implemented by the open-source RTXI^43^ Linux software—the onset of the stimulation is determined by ongoing neural events and delivered transiently through software control. We studied the effect of CW-NIR illumination focused on neurons with spontaneous tonic firing. Combining experimental results with modeling analysis allowed exploring the candidates that can explain the observed neuromodulation. We present a novel procedure for NIR laser stimulation to dissect and intervene in the waveform dynamics through activity-dependent stimulation. By interlacing results from theoretical simulations and sustained and activity-dependent stimulation, we identify the dynamical elements behind AP dynamics under CW-NIR modulation. We discard any single candidate of the biophysical effect as the joint experimental and model analyses indicate that laser illumination affects multiple membrane factors simultaneously.

## 2 Materials and Methods

### 2.1 Intracellular Recordings

The experimental data were obtained using intracellular electrophysiological recordings with sharp electrodes in the neurons of *Lymnaea stagnalis*. This animal model was used because of the easy accessibility of the neurons that are distributed and organized in different ganglia, with a distinct function associated to each one of them. For the experiments, we chose the right parietal ganglion (RPG) since it is one of the largest ganglia in the system and so are its neurons. In addition, many neurons in this ganglion are usually spontaneously active and present a tonic spiking that can last for hours. It is also convenient of this animal that its neural activity is slow, which allows us to explore the modulation of the AP dynamics at multiple time scales with larger resolution in the electrophysiological recordings. Membrane potential was recorded using 3 M KCl filled electrodes and a DC amplifier (ELC-03M, NPI Electronic, Hauptstrasse, Tamm, Germany). Recordings were acquired at 10 KHz using an A/D board (PCI-625 with a BNC-2090A DAQ device, National Instruments).

### 2.2 *Lymnaea stagnalis* Preparation

**Fig. 1:**
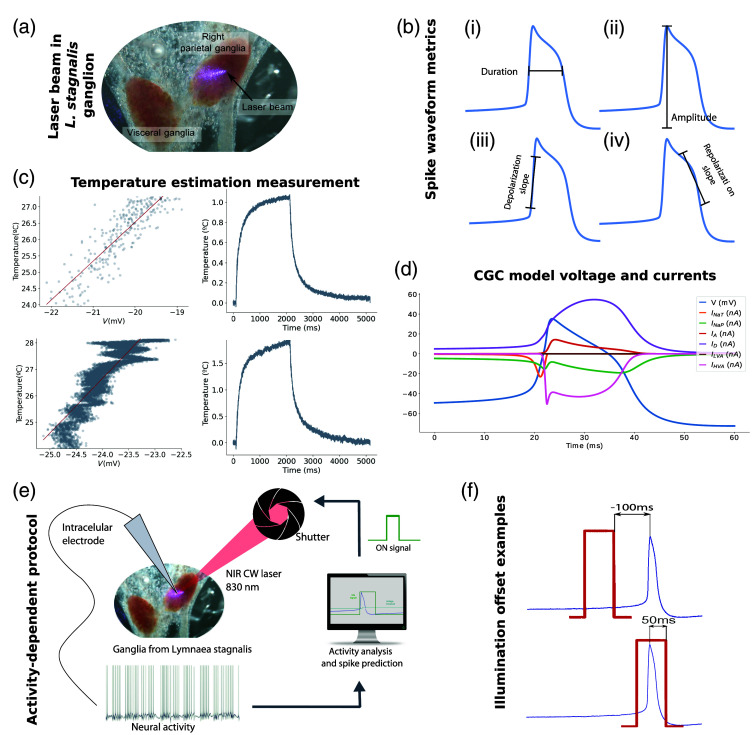
(a) Illustration of the laser beam focused on a neuron in the right parietal ganglia at maximum power showing a sharpened form due to the angle. (b) Representation of waveform shape’s metrics: (i) Spike duration at half width. (ii) Spike amplitude between the maximum and minimum voltage values. (iii) Depolarization slope at half width. (iv) Repolarization slope at half width. (c) Open-pipette temperature estimation method. Each row in the panel represents pulsed and continuous current delivery for the estimation, respectively. For both examples: left column, temperature and voltage relation. Right column filtered mean of voltage recordings from short illumination intervals in the pipette. (d) Simulation of the CGC-model representing the voltage dynamics during an action potential and the corresponding ionic currents defined in the model ($I_{\mathrm{NaP}}$, $I_{\mathrm{NaT}}$, $I_{A}$, $I_{D}$, $I_{\mathrm{LVA}}$, $I_{\mathrm{HVA}}$). The units in the y axis are specified in the legend. (e) Activity-dependent protocol scheme. Neurons were recorded intracellularly and their voltage signals were processed in real-time with the RTXI software. Using the spike prediction algorithms, the shutter was triggered at the desired action potential phase illuminating the neurons. (f) Examples of illumination offset, defined as the time interval from the end of the illumination to the peak of the spike.

The neural system was isolated from the rest of the body to perform the electrophysiological recordings. The body shell was removed, along with other visceral parts above the neural system. Then, all nerves attaching the system to the body and the buccal ganglia to the buccal mass were cut, so the system was isolated [see Fig. 1(a)]. The preparation was immersed in a saline solution (in mM: 51.3 NaCl, 1.7 KCl, $1.5\;{\mathrm{MgCl}}_{2}\cdot 6H_{2}O$, $4.1\;{\mathrm{CaCl}}_{2}\cdot 2H_{2}O$, 5 HEPES, corrected to pH 7.8 with 4 MNaOH). The sheath above the ganglia was reduced using protease (Sigma type XIV) to facilitate access to the neuron with the sharp electrode. All procedures followed the European Commission and Universidad Autónoma de Madrid animal treatment guidelines.

### 2.3 Spike Waveform Characterization Parameters

For both experimental recordings and model simulations, APs were detected as the maximum point over a threshold, and each waveform was segmented 100 ms before and after the peak temporal reference. For the superposition of APs (Figures in Secs. 3.1 and 3.2), the waveforms were aligned in the x axis by the peak and in the y axis by the first point of the waveform voltage values.

**Fig. 2:**
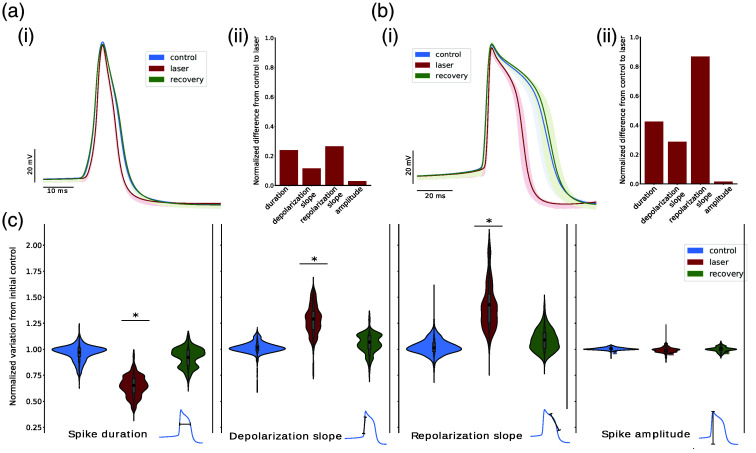
Effect of sustained CW-NIR laser stimulation on the spike waveform for two distinct neuron types. For all panels: control, laser, and recovery are color-coded in blue, red, and green, respectively. (a) Characterization of no shoulder-shaped-type neuron. (b) Characterization of shoulder-shaped-type neuron. (ai) and (bi) Superimposition of spike waveforms in a recording corresponding to a symmetrical and shoulder spike neuron, respectively. The spikes were aligned to the peak for the x axis and to the onset for the y axis, the mean is depicted in darker colors. (aii) and (bii) Bar charts quantify the change using the difference from laser to control normalized by the mean control value for metrics: duration, depolarization, and repolarization slopes, and amplitude. (c) Violin plots representing the variation of the experiments with respect to the control (N=23) for shoulder and symmetrical types together. For each metric of the waveform, the control, laser, and recovery recordings are normalized to the first control. From left to right: duration, depolarization slope, repolarization slope, and amplitude. Asterisks over the violins indicate that the metric change was highly significant [Bonferroni correction, (ρ<0.01/4), see Sec. 2.7].

For the waveform shape characterization, we used four metrics: duration, amplitude, depolarization, and repolarization slopes. They are depicted in Fig. 1(b) and defined as follows:

• Duration: Time interval between the two points at half width of the AP.
• Amplitude: Difference between minimum and maximum voltage values in the waveform in the analyzed segment.
• Depolarization slope: Slope in the depolarization phase (previous to the peak) measured 1 ms before and after the half width point reference.
• Repolarization slope: Slope in the repolarization phase (after the peak) measured 1 ms before and after the half width point reference.

These metrics were used for the quantitative analysis of the change in experimental recordings and model simulations. In Sec. 2.7, we describe the quantification methodology for the waveform metric change as well as for the comparison between the experimental and model results.

### 2.4 Temperature Estimation

To estimate the CW-NIR laser induced temperature change, we used the open-pipette method employed in previous experimental studies to measure the temperature variation during the illumination.^15^^,^^44^^–^^46^ We calibrated the resistance and temperature relation using a thermistor (EPCOS, 10  kΩ) to measure the temperature in the preparation solution in the range from 23°C to 29°C. We used two protocols: injecting a constant current to calculate the resistance change from the voltage recording and injecting pulses of a specific current value. From the resulting recording slope of the linear regression, we computed the conversion from voltage to temperature. For the estimation of the temperature change during the laser stimulation, we measured the voltage change during short intervals of laser illumination and the temperature value at its saturation plateau. This estimation is represented in Fig. 1(c).

### 2.5 Continuous-Wave NIR Laser Stimulation

The experimental results presented here were obtained using a CW-NIR diode laser in single TEM00 operation and 830 nm wavelength output (Integrated Optics 0830L-13A-NI-PT-NF). The diode laser output was coupled to a single-mode optical fiber to efficiently guide the laser beam to the sample. To adapt the divergence of the laser beam to the fiber optic output, an aspherical lenses-collimator (Thorlabs, F280FC-850) was installed. An achromatic doublet with focal length f=50  mm was used to focus the laser beam on the sample (Thorlabs AC127-050-B-ML). The experiments were performed with a laser output power of ∼90  mW and a power density over the sample of $146\;W/{\mathrm{cm}}^{2}$. The grazing incidence of the laser beam on the sample created a quasi-elliptical spot, with a minor axis of ∼34  μm, as shown in Fig. 1(a).

The laser was attached to a micro-manipulator (Siskiyou MX160), allowing micrometer precision of the beam placement over the neuron and optimization of the beam focus. The focusing was performed using a binocular microscope (Nikon SMZ-1500) coupled to a CCD camera (XCAM1080PHA, ToupTek Photonics, Zhejiang, China).

For the activity-dependent stimulation experiments described below, the CW-NIR laser light was blocked with a mechanical shutter (Thorlabs SH05, Newton, New Jersey). The shutter was triggered by a TTL signal upon the real-time detection of neuronal voltage events [see Sec. 2.9 and Fig. 1(e)]. The shutter utilized in this work had a ∼8  ms delay from the trigger signal, which might be a limitation for neural stimulation in fast spiking cells. The slow spike dynamics in the neurons used for this research are compatible with this restriction, and the protocol was developed considering this limitation.

### 2.6 Sustained CW-NIR Stimulation Protocol

After the isolation of the *Lymnaea stagnalis* neural system, we searched for suitable neurons in the RPG, i.e., cells with spontaneous activity preferably with fast activity and shoulder or symmetrical type in the spike waveform. Once the target neuron was identified, the laser was set-up. A lens with focal distance of f=50  mm was used and no polarizer was installed on the optical path. Guided with the microscope camera, the laser spot was located and then placed first over the ganglion and subsequently over the specific neuron where the electrode was recording the activity. At this point, the laser was focused with the micro-manipulator adjusting the focal distance. Finally, the laser power was increased to the above mentioned value for the experiments.

Once the laser spot was over the neuron while the membrane voltage was simultaneously recorded at the soma with the intracellular electrode, we followed the protocol described below to measure the effect of the CW-NIR laser on the activity of the neuron:

1. First control. The spontaneous activity in the neuron was recorded for 1 to 3 min, depending on the spiking frequency of the cell. During this control, there was no external modulation of the neuron apart from the possible alteration by the intracellular procedure.
2. Laser stimulation. The laser was on during the same lapse of time than in the first control, stimulating the neuron with a constant optical power density. There was no modification in laser parameters during this time.
3. Recovery. After the illumination was off, a second control was performed, under the same conditions as the first one. During this recovery control, the activity in the neuron after the effect of the laser was recorded.

The sequence involving control, laser, and recovery trials was replayed in each experiment (day and individual) for five times. Between each trial, the laser illumination was supervised to ensure that the spot was still over the neuron, guaranteeing that the procedure had as low variation as possible. Also, the laser was only turned off during the controls, it was not set aside, since that would have forced to redo the set-up for every trial altering the reproducibility between trials. The effect for each trial of a given day was very similar. For the analysis in Sec. 3.1, the trial with the strongest effect in the day was selected.

### 2.7 Statistical Analysis

The statistical significance analysis in the data obtained from the sustained CW-NIR stimulation protocol in Sec. 3.1 was performed applying a paired T-test to the four spike waveform metrics characterized here [see Fig. 1(b)]. Data from distinct experiments were gathered and paired by recordings of control-laser and control-recovery. The null-hypothesis tested was that control group was equal to the laser group and that the control group was equal to the recovery group, respectively. Since we performed the test in the four waveform metrics—spike duration, depolarization slope, repolarization slope, and amplitude—we applied the Bonferroni correction, thus we considered high significance when ρ<0.01/4.

To compare recordings from different neurons, we normalized the change between the laser and the control conditions for each waveform metric using the following expressions for electrophysiological data:

$${\text{metric change}}_{\text{experimental}}=\frac{\left|\mu ({\text{metric}}_{\text{laser}})-\mu ({\text{metric}}_{\text{control}})\right|}{\left|\mu ({\text{metric}}_{\text{control}})\right|},$$ (1)

where $\mu ({\text{metric}}_{\text{laser}/\text{control}})$ is the mean of the corresponding metric for all waveforms in a given laser stimulation or control trial.

Analogously, to compare the change between the distinct model simulations, we normalized the change in the parameter-driven simulated variability range using the following expression:

$${\text{metric change}}_{\text{model}}=\frac{{|\text{metric}}_{\min}{-\text{metric}}_{\max}|}{{|\text{metric}}_{\max}|},$$ (2)

where ${\text{metric}}_{\min/\max}$ refers to the minimum or maximum value of the corresponding waveform metric resulting from the model simulations in the considered parameter range.

For the comparison between experimental data and model simulations in Sec. 3.2, we defined an experimental reference for each metric as the general mean and standard deviation for all experiments (N=23). This allowed us to define a range of change due to the laser effect to which the model values could be compared. The mean of metric experimental change (MEC) was defined as

$$\mu_{\mathrm{MEC}}=\frac{1}{N}\overset{N}{\underset{i=1}{\sum }}\frac{{\left|\mu {\left(\text{metric}\right)}_{\text{laser}}-\mu (\text{metric}{)}_{\text{control}}\right|}_{i}}{{\left|\mu (\text{metric}{)}_{\text{control}}\right|}_{i}}.$$ (3)

Here, $\mu (\text{metric}{)}_{\text{laser}}$ and $\mu (\text{metric}{)}_{\text{control}}$ represent the mean values for each experiment i in laser stimulation and control trials, respectively, where the index i ranges for all the experiments, with N being the number of experiments.

Thus, the percentage change in waveform from the model simulations, as described in Eq. (2), was mapped to this reference range: $\left(\mu_{\mathrm{MEC}}\pm 2\sigma_{\mathrm{MEC}}\right)$, with $\sigma_{\mathrm{MEC}}$ being the standard deviation of the MEC. To visually represent this range, we utilized a color gradient with the *background_gradient* option in *DataFrame* style in Python. The equation for mapping these values is

$$\text{Gradient value}=\frac{\text{value}-V\min}{V\max-V\min}.$$

Here, value represents the percentage change in the model simulation for a specific metric, while Vmin and Vmax represent $\mu_{\mathrm{MEC}}-2\sigma_{\mathrm{MEC}}$ and $\mu_{\mathrm{MEC}}+2\sigma_{\mathrm{MEC}}$, respectively, with $\mu_{\mathrm{MEC}}$ and $\sigma_{\mathrm{MEC}}$ being the specific mean and standard deviation corresponding to the metric specified in value. Since all changes are in absolute value, the lower bound for Vmin is 0.

All data analyses were performed in Python, the scripts are available in a GitHub repository at: https://github.com/GNB-UAM/Garrido-Pena_Modulation-neural-dynamics-by-CW-NIR-stimulation.

### 2.8 Neuron Models

The theoretical study was carried out simulating the laser modulation on the neurons in three conductance-based models: (i) the classic Hodgkin–Huxley model^47^ defined by Eq. (4), composed of two active ionic channels: $I_{\mathrm{Na}}$ and $I_{K}$, and a leakage current; (ii), a N3t neuron model,^48^ with two compartments and defined by Eqs. (5) and (6), which represents a neuron in the *Lymnaea stagnalis*’s feeding CPG,^49^ and (iii) a model from *Lymnaea stagnalis*, which simulates the CGC neuron located in the cerebral ganglia with a shoulder type spike waveform.^50^ This model is described by six different ionic channels: persistent and transient sodium currents ($I_{\mathrm{NaP}}$, $I_{\mathrm{NaT}}$), transient and delayed rectifier potassium currents ($I_{A}$, $I_{D}$), and low-voltage-activated and high-voltage-activated calcium currents ($I_{\mathrm{LVA}}$, $I_{\mathrm{HVA}}$), described by Eqs. (713). The CGC neuron model is the one used for the temperature dependence study since it has the most detailed description in terms of combination of channels. Figure 1(d) shows the dynamics of each channel in this model during an AP:

$$C_{m}\frac{dV}{dt}=I_{\mathrm{inj}}-I_{\mathrm{Na}}-I_{K}-I_{L},$$ (4)

$$\tau_{m}\frac{dV_{S}}{dt}=i_{\mathrm{inj}}-i_{L,S}-i_{T}-i_{ec,S}-i_{\mathrm{syn}},$$ (5)

$$\tau_{m}\frac{dV_{A}}{dt}=-i_{L,A}-i_{\mathrm{NaT}}-i_{K}-i_{ec,A},$$ (6)

$$C_{m}\frac{dV}{dt}=I_{\mathrm{inj}}-I_{\mathrm{NaT}}-I_{\mathrm{NaP}}-I_{A}-I_{D}-I_{\mathrm{LVA}}-I_{\mathrm{HVA}},$$ (7)

$$I_{\mathrm{NaT}}=g_{\mathrm{NaT}}m_{\infty }^{3}h(V-E_{\mathrm{Na}}),$$ (8)

$$I_{\mathrm{NaP}}=g_{\mathrm{NaP}}r^{3}(V-E_{\mathrm{Na}}),$$ (9)

$$I_{A}=g_{A}a^{4}b(V-E_{K}),$$ (10)

$$I_{D}=g_{D}n^{4}(V-E_{K}),$$ (11)

$$I_{\mathrm{LVA}}=g_{\mathrm{LVA}}c_{\infty }^{3}d_{\infty }(V-E_{\mathrm{Ca}}),$$ (12)

$$I_{\mathrm{HVA}}=g_{\mathrm{HVA}}e^{3}f(V-E_{\mathrm{Ca}}).$$ (13)

#### 2.8.1 Temperature dependence in the model

To simulate the temperature dependency in the neuronal activity, a $Q_{10}$ factor was incorporated to every dynamical equation in the model (i.e., conductances and activation gates). $Q_{10}$ represents the temperature sensitivity in each channel, and it was included as a new factor as shown in Eqs. (14) and (15), with $i={\mathrm{Na}}_{T},{\mathrm{Na}}_{P},A,D,\mathrm{LVA},\mathrm{HVA}$ for Eq. (14) and i=h,r,a,b,n,d,e,f for Eq. (15). The capacitance was also defined as temperature dependent ($C_{T}$) with a linear relation to the difference of temperature in Eq. (16):

$$g_{i}(T)={\overset{¯}{g}}_{i}Q_{10}^{i\frac{T-T_{0}}{10}},$$ (14)

$$\phi_{i}(T)={\overset{¯}{\phi }}_{i}Q_{10}^{i\frac{T-T_{0}}{10}},$$ (15)

$$C_{T}=c_{0}+c_{0}\gamma (T-T_{0}).$$ (16)

where ${\overset{¯}{g}}_{i}$, ${\overset{¯}{\phi }}_{i}$, $c_{0}$ are the original values used in the model and γ=0.05.

### 2.9 Activity-Dependent Laser Stimulation Protocol

For stimulating the neurons depending on their ongoing activity, a closed-loop protocol was designed in the RTXI real-time software.^43^ This hard real-time tool allows an easy integration of new modules to read ongoing activity, process it online and send feedback in the form of analog signals. The real-time module designed for this experiment followed the scheme shown in Fig. 1(e). After processing the signal in RTXI, a TTL pulse was sent to the controller opening the laser shutter for the desired time, thus stimulating the neuron during that time interval. Simultaneously, the neural activity was recorded, along with the TTL and the shutter feedback (recording the shutter delay with respect to the on signal).

The main challenge in this protocol is the identification of the specific phase of the spike waveform to deliver the stimulus, i.e., predicting the spike, since spontaneous neural activity has intrinsic variability that requires the online adaptation of specific thresholds instead of a hand-tuned preset value. For this purpose, the protocol relied on the reference values from the previous spike at a specific time interval from the peak. The voltage threshold was calculated based on the voltage value measured in the previous spike and recalculated at each action potential. Thus, after each spike, the threshold for the spike prediction was updated using the following equation:

$$V_{\text{threshold}}=V[t_{\text{spike}}[i-1]-\tau ],$$ (17)

where τ is the selected time for the prediction before the spike and $t_{\mathrm{spike}}$ is the time instant of the spike peak.

This prediction is effective for stereotyped spikes when only low amplitude subthreshold oscillations occur, but it is limited in other scenarios. Therefore, for neurons or action modes of the same neuron when it was necessary to stimulate before the depolarization rise, another reference was used: the area of the recorded voltage. In this other mode of the protocol defined in Eq. (18), the voltage was accumulated along the activity and the sum was reset after each spike:

$$V_{\text{area}}=\int_{V[{\text{spike}}_{i-1}]}^{V[{\text{spike}}_{i}]}V(t).$$ (18)

The stimulation was triggered when the area reached a specific threshold, which was predicted as in the voltage case, or hand-tuned. For the detection of the minimum point to reset the voltage area, we used an RTXI module based on RTHybrid, a real-time software that includes automatic adaptation algorithms to handle the ongoing variability of the recordings^36^^,^^37^^,^^40^ available in a GitHub repository at: https://github.com/GNB-UAM/rthybrid-for-rtxi/tree/master/rthybrid_burst_analysis. The use of each mode of the protocol in the experiment was decided depending on the specific requirements of the recorded activity.

Using this detection protocol, we assessed the effect of the illumination at different phases of the AP in the range from 100 ms before the spike peak to 80 ms after the spike peak. The illumination interval was 58 ms, validated as the minimal duration for effective stimulation in the test trials. The RTXI module programmed for this study is available in a GitHub repository available at: https://github.com/GNB-UAM/spike_predictor.

## 3 Results

### 3.1 Sustained CW-NIR Laser Stimulation Effect on Single Neuron Dynamics

#### 3.1.1 CW-NIR laser effect on spike waveform

In this paper, we performed experimental triplets of control, sustained CW-NIR laser stimulation and recovery recordings (for details see Sec. 2.6). This protocol provided a reference for the characterization of the laser effect. The data analyzed in this section correspond to the spontaneous activity of neurons from the RPG of *Lymnaea stagnalis*, under no stimulation other than the laser illumination when specified.

Left panels in Figs. 2(a) and 2(b) illustrate the stereotyped waveform of the AP from two experiments in two distinct neuron types present in the RPG with symmetrical and shoulder spike shapes, respectively. Note that the two neuron types differ not only in spike waveform but also in duration. In the example shown in Fig. 2(a), the duration of the spike was ∼20  ms whereas the one shown in Fig. 2(b) was ∼40 to 50 ms. To characterize the sustained CW-NIR laser stimulation in terms of change and recovery, the three stages of the protocol—control, laser, and recovery—are represented in all panels. The superimposition of the spike waveforms (∼40 and 110 spikes for each trial, panels (a) and (b), respectively) for the same recording are aligned in the x axis by the spike peak and in the y axis by the voltage amplitude of the first point of the waveform, together with the trial mean spike represented with a wider line. Note how the control and recovery traces overlap for both neuron types, illustrating the resumption of the spike dynamics shortly after the laser stimulation ceases [see aligned spikes in Figs. 2(a) and 2(b)].

Figures 2(a) and 2(b) illustrate that the variability was very small in amplitude, duration, and in depolarization or repolarization slopes between the spikes within the same trial in both neuron types. However, during CW-NIR laser stimulation, the change in AP waveform shape was notable with respect to the control and the recovery. This change was most clear in the spike duration, which was the result of changes in both depolarization and repolarization slopes.

The right panels (ii) in Figs. 2(a) and 2(b) show bar charts that quantify the change in terms of spike duration, amplitude, depolarization slope, and repolarization slope. These metrics were used to characterize the AP waveform and its possible change during the laser illumination [see also Fig. 1(b) and Sec. 2.3]. Each one of these metrics is represented on the right panels as the absolute value of the difference of the laser stimulation to the control recording normalized by the mean control value (see Sec. 2.7). For both neuron types, there was a change in duration and in the slopes, with the largest change being in the repolarization slope (around 26% for the symmetrical spike type neuron and 86% for the shoulder type). The alignment illustrated in the left panels shows that the change in amplitude was minimal in comparison to the rest of the metrics. Although both neuron types showed an effect of the sustained CW-NIR laser stimulation in the AP waveform, the change in the shoulder neuron type was larger for duration and slopes. This may be due to specific channels that generate the shoulder shape of the spike, which may allow for a wider range of change in the spike dynamics, especially in terms of the repolarization slope.

Figure 2(c) displays the results of multiple experiments following the same protocol described above, represented in violin plots as the normalization of each experiment with respect to the mean of the first control of the respective metric for each spike detected during control, laser, and recovery. To avoid possible bias from the natural evolution of the intracellular recordings, in this figure, we only included experiments where the activity was recovered within 10% change in firing rate with respect to the control. For each trial, only stereotyped waveforms were considered and large deviations (in the form of $z_{\mathrm{score}}<-0.1$ in the normalized duration) were filtered out. The variability characterized in the control violins represents the variation within controls, which was also the most homogeneous in terms of density distribution. This is represented for each one of the selected spike waveform characterization metrics as in Figs. 2(a) and 2(b)—duration, depolarization slope, repolarization slope, and spike amplitude [see Fig. 1(b)].

The results shown in Fig. 2(c) are consistent with the described change in the illustrative individual experiments shown in Figs. 2(a) and 2(b) in the same figure. On the one hand, the activity recovered its initial characteristics after the CW-NIR laser stimulation ceased for every metric, i.e., the recovery (green violins) returned to the same level as the control (blue violins). The differences in these distributions are mainly caused by the natural variability in the biological system. Also, as all values are normalized to the mean of the first control, it can be expected that the distributions may diverge more in laser and recovery violins than in control violins. A separate analysis for the two neuron types is available in Fig. S1 in the Supplementary Material.

Regarding the change during the laser stimulation, for every waveform metric except amplitude, we can see in Fig. 2(c) how the overlapping of the distributions is minimal. The distribution for the duration was the most homogeneous, whereas the variation for depolarization and repolarization slopes had different density distributions, being the repolarization slope the one presenting a larger change in most cases. This can be explained by the variety of neurons in the collected data, the change in the slopes differed from one type of neuron to another. Thus, the distribution of variability was different. Some laser stimulation recordings presented a milder change than others. The slight change along neurons of the same type was likely due to the physical restrictions of the setup in each experiment: the angle of the laser, the laser focusing, the maximum power used, and the overlaying tissue. Overall, considering these factors, we can see that stimulating with the sustained CW-NIR laser resulted in a significant change of the spike waveform. In the case of the amplitude, the change was very small.

We performed statistical tests on these data and confirmed that the changes in duration, depolarization, and repolarization slopes were highly significant (ρ<0.01/4) when comparing control and laser samples. The amplitude change was not highly significant, and so were not the changes in any metric comparing control and recovery samples (see Sec. 2.7).

This combination of changes points out different biophysical candidates that might be involved in the modulation of the global change in both slopes or specific channels involved in the CW-NIR laser effect, since the depolarization and repolarization slopes were affected differently, while the amplitude did not change, and for distinct neuron types, the characterized metrics had different variations (i.e., the repolarization slope in the shoulder-shaped neuron type was reduced to a greater extent than in the symmetrical type). In Sec. 3.2, we assess these possible candidates using a computational model.

#### 3.1.2 CW-NIR laser effect on spiking rate

During the identification of the biophysical effect at different phases of the AP dynamics on single neurons, we identified a robust acceleration of the AP (a shorter duration of the spike waveform). This could also point to an acceleration of the tonic activity of the neurons. Pulsed NIR laser stimulation has been proven effective as a stimulation technique, mainly eliciting APs in silent neurons at specific combinations of pulse duration and intensity.^11^^,^^12^^,^^17^^,^^18^ Thus, we also assessed the effect during sustained CW-NIR infrared laser stimulation on the spiking frequency in long stimulation recordings (1 to 3 min).

To avoid possible bias originated from intrinsic properties of the neuron and the circuit in which it was integrated, we only considered recordings where the neurons effectively recovered their control activity rate after the stimulation (i.e., absolute recovery change within 10% from the initial control). The activity frequency was characterized by the absolute firing rate (AFR) for control, laser, and recovery, and by a histogram of inter-spike intervals (ISIs), i.e., the time interval from peak to peak.

**Fig. 3:**
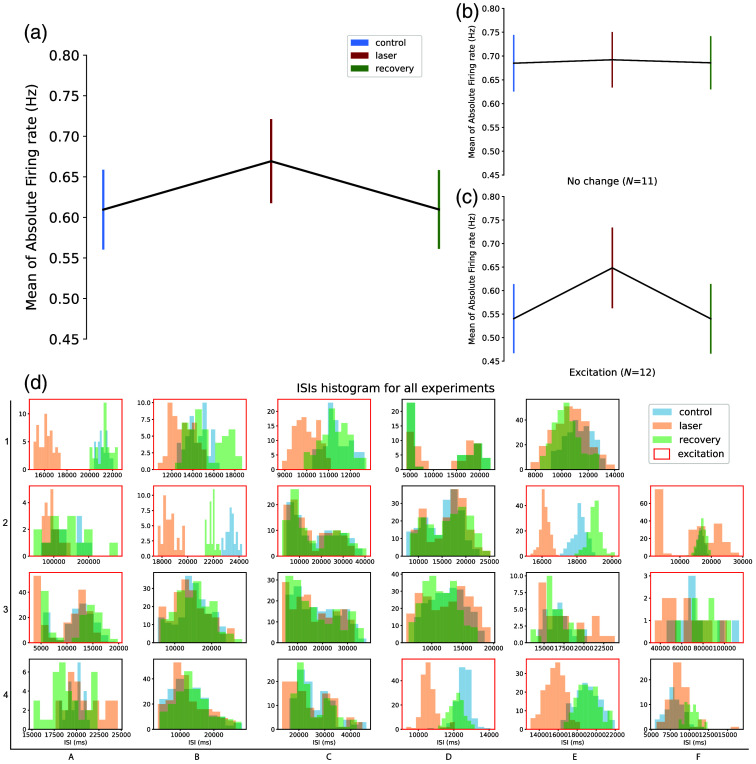
Firing rate and ISIs analysis for the CW-NIR laser stimulation. (a) AFR in all experiments (N=23). (b) AFR for cases from the experiments in (a) with no change during laser illumination (N=11); (c) AFR for experiments from (a) showing an increase in the firing rate (N=12); (d) ISI histograms for control, laser, and recovery for each experiment (blue, red and green, respectively). Cases showing increased excitation in their firing rate (sample in c) when illuminated by the CW-NIR laser are highlighted in a red square.

In Fig. 3, the mean firing rates for control, laser, and recovery are represented along with their standard error of the mean. Figure 3(a) depicts the general change in frequency for the neurons, showing the neural activity trend to excitation in the mean. In Figs. 3(b) and 3(c), this set of triplets is divided into two groups depending on the difference between the laser and the control, classified as no change when the difference between the control and laser was less than 10% [Fig. 3(b)], and as excitation for the opposite case [Fig. 3(c)]. There is no representation of inhibition in this panel, since there was not any experiment that fulfilled the criteria of a 10% negative change during the laser stimulation with respect to the control. Note how cases where the activity increases are the most consistent ones (12 out of 23) and that even in the set classified as unchanged, the mean of the AFR during laser stimulation is larger than the controls. These results support an excitatory tendency during CW-NIR sustained stimulation.

The AFR hinders some characteristics of the neural activity, such as the refractory period or the presence of bursting activity, which might also influence the firing frequency study. Thus, Fig. 3(d) displays the ISI histogram for each experiment showing again the triplets of control, laser, and recovery, for each sample. Experiments showing excitation are highlighted in a red square. Note that for most cases classified as excitation, the ISIs tendency is to be reduced, which is observed in the laser histogram at the left of the control and the recovery. Note that there are some experiments where the laser ISI histogram seems to overlap with the controls and the recovery [see Fig. 3(d), subpanels (2,A), (3,A), and (2,C)] but still the mean AFR of the laser recording was 10% higher than the control. In these situations, even though the activity was faster under stimulation, the time between spikes did not show a proportional change, which can be due to a modulation in the refractory period that compensated for the spike acceleration. Under the laser modulation, we also found that some neurons would start firing in shorter ISIs, tuning the tonic spiking into pair spiking similar to small bursts, e.g., Fig. 3(d) and [2,F].

Overall, our results in this subsection show a larger tendency to a frequency increase in response to the NIR illumination indicating that it is possible to achieve neuronal excitation under sustained CW-NIR laser stimulation. It is also important to highlight that inhibition was not found in any of these experiments with sustained CW-NIR laser stimulation during tonic firing activity.

### 3.2 Model Analysis for Constraining Candidates to Explain the Effect of CW-NIR Laser Illumination in the Spike Dynamics

Computational models are a powerful tool to assess the source of neural dynamics where all variables involved are accessible. By considering membrane potential recordings alone, it is difficult to understand the contribution of the biophysical candidates in the underlying dynamics shaping the AP. While it is especially hard to carry out experiments using chemical and/or electrophysiological techniques to selectively block or compensate channels to mimic the observed CW-NIR laser effect, the simultaneous accessibility to all the variables in a model provides a unique tool to dissect the contribution of all biophysical candidates. Thus, to further explore the source of the experimentally observed CW-NIR effect, we analyzed the spike generation dynamics in three different conductance-based models assessing the change in the most likely candidates to be affected by the laser stimulation: modulation of membrane capacitance and ionic channels. More specifically, we modulated (i) the capacitance and the conductance of the active ionic channels—$I_{\mathrm{Na}}$ and $I_{K}$—in the standard Hodgkin–Huxley (HH) model^47^; (ii) the conductance of ionic channels—$I_{\mathrm{NaP}}$, $I_{\mathrm{NaT}}$, $I_{D}$, $I_{A}$, $I_{\mathrm{HVA}}$, $I_{\mathrm{LVA}}$—and capacitance in a *Lymnaea stagnalis* CGC neuron model with a shoulder-shaped waveform;^50^ and (iii) the capacitance in a two-compartment model—where the fast and slow dynamics are segregated—in a *Lymnaea stagnalis* buccal ganglion neuron (N3t) model.^48^ The implementation of these models is available in the open-source model library Neun in a GitHub repository at: https://github.com/GNB-UAM/neun and the code for the simulations can be accessed in a GitHub repository at: https://github.com/GNB-UAM/Garrido-Pena_Modulation-neural-dynamics-by-CW-NIR-stimulation.

The model parameters were modulated to investigate and compare their effect to that of the CW-NIR laser stimulation on the neurons, and to evaluate the interrelationship between the observed changes. To identify changes in the spike dynamics similar to those observed under the CW-NIR laser illumination, in this section, we covered a complete range of values in the parameter space of each biophysical candidate. The criteria for driving the parameter exploration were the preservation of tonic spiking in the activity and the assessment of a realistic range of values. As our initial hypothesis did not assume that the CW-NIR laser effects were exclusively photo-thermal, model parameter changes were applied with no temperature description. Further down in Sec. 3.2.3 we present a detailed study considering the temperature dependency of the biophysical candidates.

**Fig. 4:**
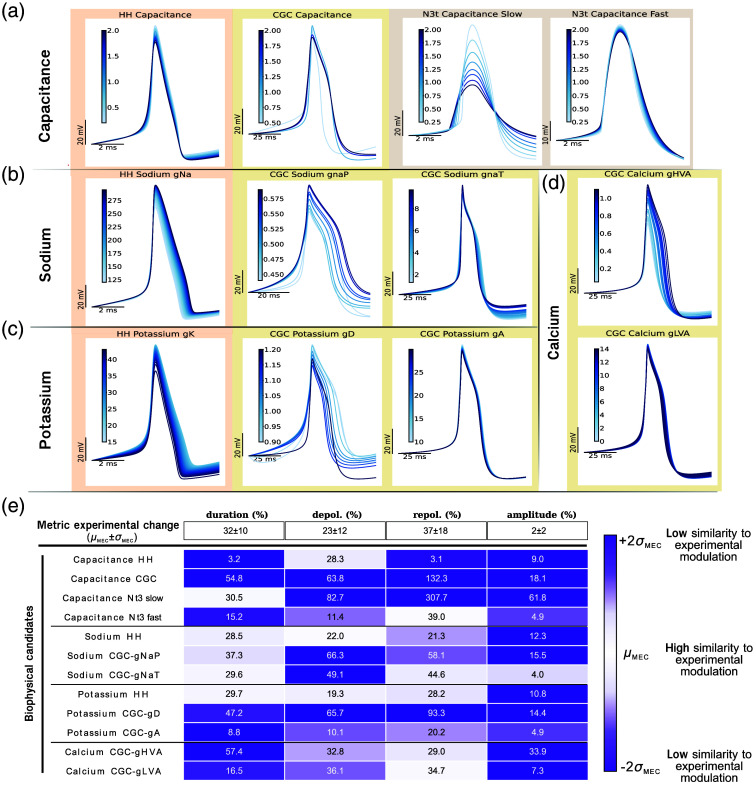
Modeling study of the CW-NIR laser stimulation effects due to isolated biophysical changes that alter the spike waveform. (a)–(d) Superposition of spike waveforms in each model by modulating a single biophysical candidate. The background colors correspond to each simulated model. In (a), the capacitance is changed for the HH and CGC neuron models, and in the two compartments for the N3t neuron model. (b) The spike waveforms changing the conductances of Na channel currents: $I_{\mathrm{Na}}$ from the HH-model, $I_{\mathrm{NaP}}$ and $I_{\mathrm{NaT}}$, from the CGC-model. (c) The modulation of K conductances in ionic currents: $I_{K}$ in the HH-model and $I_{D}$ and $I_{A}$ for the CGC-model (from left to right). (d) The modification of the calcium current conductances in the CGC-model ($I_{\mathrm{HVA}}$ and $I_{\mathrm{LVA}}$). Table in (e) represents the quantification of the changes in the spike metrics when tuning each parameter for every model. Each cell contains the waveform change normalized to the maximum. The color gradient represents similarity based on the standard deviation of the normalized experimental change. Dark purple corresponds to low similarity ($2\sigma_{\mathrm{MEC}}$ or larger) and white to high similarity. The quantified experimental reference (MEC) is annotated in the first row of the table.

The results of this study are shown in Fig. 4. The analysis for each model is sorted by the explored biophysical candidate—capacitance, sodium channels, potassium channels, and calcium channels. Thus, Figs. 4(a)–4(d) show the superposition of all spike waveforms from the simulations for the range of explored model parameter values of each candidate. The table in Fig. 4(e) represents how well the different model candidates reproduce the observed experimental effect. For each metric and biophysical candidate, there is a percentage of change in the model calculated as the change from minimum to maximum normalized with the maximum value (analogously to the quantification in Fig. 2, see also Sec. 2.7). The background color in each cell represents the ability of each model parameter modulation to produce results similar to the change in the experimental results. The color gradient (represented in the color bar) takes as reference the mean of the MEC quantification, considering the range of $\left(\mu_{\mathrm{MEC}}\pm 2\sigma_{\mathrm{MEC}}\right)$ (see Sec. 2.7). The mean change and its standard deviation were computed as the normalized difference between mean values for each control and laser experimental pair for all experiments. These reference values are shown for each metric in the first row of the table in Fig. 4(e) to compare them with the model results. Thus, dark purple corresponds to values two times the STD of the mean over or under the mean, and white represents the midpoint between those two values, i.e., high similarity to the experimental modulation. For example, in the case of capacitance in the HH model, the change in duration was minimal 3.2%, while the mean change in the experimental observation was 32%, this color is then represented in dark purple, since 3.2% is not in the defined range (32±20). On the other hand, the change in depolarization slope for this model (28.3%) is depicted in light purple, since it is in the defined range and close to the mean MEC (23±12).

#### 3.2.1 Change in capacitance

Capacitance has been one of the most discussed biophysical candidates to be affected by IR laser illumination.^17^^,^^18^^,^^26^^,^^51^ A change in capacitance has a direct effect on the spiking frequency and exerts a global modulation on all ionic currents, so many studies have discussed this change both experimentally and theoretically. Here, we explored the CW-NIR modulation of capacitance in three different conductance-based models: in the Hodgkin–Huxley model, used in other studies, in the CGC–Vavoulis model, which presents a variety of channels, and in the N3t–model, which is the only one with more than one compartment and, thus, has two distinct capacitance values.

Figure 4(a) shows the waveforms of each simulation. In the case of the HH-model, there was a mild change in duration, mainly caused by the depolarization modulation, and a change in amplitude larger than what was observed experimentally. The CGC-model exhibited a similar tendency to the HH-model but with an extreme case at a low value of capacitance $0.5\;\mu F/{\mathrm{cm}}^{2}$, where moderated changes in depolarization and amplitude were combined with a large change in the repolarization, and consequently in the duration. This modulation made capacitance a better candidate for reproducing the experimental results in the case of the CGC neuron, preserving the metrics’ change interrelations, i.e., the combination of a minimal change in amplitude and a large change in duration, together with a larger change in repolarization than in depolarization slope. In the N3t neuron, we can see contrasting results for the two different compartments. In the compartment containing slow currents, the change in amplitude was the most striking, seemingly conditioned by both slopes. In the case of the fast current compartment, the main change was observed in repolarization rather than depolarization, which is more similar to the experimental outcome.

These results are quantified in the table in Fig. 4(e). The HH-model showed a change comparable to the experimental one only for the depolarization slope. The CGC-model reached plausible values in terms of the interrelation between metrics (large change in duration generated by a larger change in repolarization than in depolarization slope), but it exceeded the experimental references. The change in amplitude was larger than seen in the experimental results for most parameters (dark purple). It is especially clear in the case of the two-compartment model (N3t neuron), in which the modulation of capacity in the slow compartment resulted in a large change in amplitude. In contrast, the small change in amplitude in the fast compartment was the most similar value with respect to the experimental results (light purple).

By changing the capacitance, we achieved some of the expected changes, but achieving the desired results for all four metrics simultaneously was not possible. Therefore, modulating capacitance alone does not reproduce the experimentally observed effects, especially regarding the combined change, e.g., a large change in repolarization and a small change in amplitude, a larger change in repolarization than in depolarization. It was only when the fast compartment of N3t was modified that relations between these four metrics matched the above relationships. But note that changing the capacitance in the slow compartment is equivalent to changing several ionic currents simultaneously, not just a single current property.

#### 3.2.2 Ionic channels

The other mechanism to explain the laser modulation that we assessed here was a direct effect on the ionic channels involved in the generation of APs. These channels are activated in a sequential manner, and each of them is directly involved at distinct stages of the AP generation. They have been discussed in the laser stimulation literature^13^^,^^44^^,^^45^ by a direct effect of maximal conductance, and channel opening and closing dynamics due to thermal effects, e.g., in calcium channels.^16^^,^^27^ These candidates were assessed here in the two single-compartment models, the HH-model due to its wide use in computational neuroscience and the Vavoulis-CGC model for its variety of channels (including calcium currents) and accurate reproduction of the observed neural waveform shape. Note that in the CGC-model analysis, all current types are in pairs of high and low conductance as well as fast and slow dynamics, having two currents for sodium, potassium, and calcium [see Fig. 1(d) in Sec. 2].

In Fig. 4(b), the spikes from the simulations for each sodium current in the HH and CGC models—$I_{\mathrm{Na}}$, and $I_{\mathrm{NaP}}$ and $I_{\mathrm{NaT}}$, respectively—are superimposed. For the three currents, we observed modulation in both depolarization and repolarization slope, which resulted in a change in duration. Although the change in duration is close to the experimental outcome, the change in the depolarization is larger than in repolarization [see Fig. 4(e)], which is contrary to the experimental results, as it is also the change in amplitude for $I_{\mathrm{Na}}$, and $I_{\mathrm{NaP}}$. However, for the channel $I_{\mathrm{NaT}}$, the change in amplitude was smaller, falling closer to the experimental range for amplitude and duration, but the change in depolarization exceeded the experimental range and the repolarization change was limited in the context of shoulder type neurons (the waveform type that reproduces CGC-model). Although the change of sodium channels alone generated a similar change in duration in relation to the experimental results, the rest of the metrics did not replicate those results.

Analogously, in Fig. 4(c), simulations for potassium currents ($I_{K}$ and $I_{D}$ and $I_{A}$, respectively) are represented for HH and CGC models. For the three currents, the major change was in the repolarization slope followed by the depolarization [see quantification in Fig. 4(e)]. This combination resulted in a modulation of the duration that lay in the range of similarity to the experimental results, with the exception of the amplitude, which does not correspond to the experimental results. It is especially applicable in the case of $I_{K}$ in HH-model and in the conductance of the strong potassium current $g_{D}$ of the CGC-model. Note that for the $I_{A}$ current, although the combination of changes was comparable to the experimental change, their range was not, so a change in this current alone was not considered a plausible candidate. Thus, a change in potassium channels reproduced the experimental results for the duration and the two slopes overall, but it was limited due to an excessive change in amplitude.

In order to inspect the CGC-model in detail, we also simulated the changes in the calcium currents—$I_{\mathrm{HVA}}$ and $I_{\mathrm{LVA}}$—for this model. These currents have a key role in the generation of the shoulder shape in the spike [Fig. 4(d)]. Both created a similar change in the repolarization slopes, as well as in the depolarization, which is also close to the experimentally observed modulation. For duration, $I_{\mathrm{HVA}}$ better matched the change, but this modulation was also accompanied by a large change in amplitude which was not observed in the experimental results. On the other hand, $I_{\mathrm{LVA}}$ had one of the minimum effects on amplitude but, contrary to experimental results, its effect on the duration was also minimal, although the depolarization and repolarization slopes had a comparable change to the experimental observations. Therefore, altering each calcium channel effectively reproduced the desired change in the slopes but the modulation in duration and amplitude occurred in the same proportion, which does not match the experimental results.

The results described in this section indicate that each candidate can be modulated to bring the waveform closer to the experimentally observed results, but when changed separately they account only for a partial set of metrics matching. The desired combination of changes for duration, slopes, and amplitude was not achieved by tuning only one parameter at a time. However, some of the candidates came close to this combination. Considering the ionic current candidates, the one that was closer to the *in vivo* stimulation was potassium current, which reproduced a large range of change in the repolarization, depolarization, and duration, though exceeding the change in the spike amplitude. This is relevant in terms of maintaining the observed interrelation of the metrics. Considering the range of change reached, the calcium channels were the best candidates for the reproduction of the experimental repolarization slope modulation, allowing a wide range of values and generating the shoulder-shaped waveform. We also saw how capacitance in single-compartment models was not enough to reproduce the results. It was only when the capacitance was modified separately in two compartments, that the change reproduced the CW-NIR laser stimulation better. This points to a mechanism for explaining the CW-NIR laser effect with contribution from several candidates at the same time where specific factors might be of greater importance, such as the potassium channel in the case of shoulder-shaped neurons.

#### 3.2.3 Change of spike dynamics considering temperature modulation in the model

Most studies in laser stimulation point out to a photo-thermal effect, e.g., see Refs. 17, 24, 25, 44, 45, 52, and 53. Thus, in this section, we include a model analysis with temperature modulation. We selected the CGC-model from Ref. 50 since it is the richest model in terms of variety of channels and ability to mimic the spike waveform of shoulder-type neurons. To study global temperature dependence in the model we added a $Q_{10}$ coefficient, representing the temperature sensitivity in the model parameters. The value for this parameter is usually applied to different channel properties and kinetics in a range from 1 to 4.^54^^–^^57^ Thus, we choose 3 as a common value for $Q_{10}$, as an average general value used in the literature,^17^^,^^44^^,^^45^^,^^58^^–^^60^ and also proposed as a universal value for $Q_{10}$ to characterize temperature dependency for biochemical processes.^61^ We estimated the temperature change under laser stimulation at maximum power following an open-pipette method, with a resulting temperature increase of 1°C to 2°C [see Sec. 2.4 and Fig. 1(c)]. Note that our CW-NIR laser wavelength is at one of the lowest absorption bands of water, so the open-pipette method probably underestimates the change in temperature in the neuron, being the change in the temperature caused not only by water heating but also by heating the tissue. In addition, the reported temperature range of laser-induced variation in the literature is wide, depending on the system, the estimation technique and whether it comes from a model or an *in vivo* estimation.^17^^,^^45^^,^^62^ Therefore, in our simulations, we explored a wider range than our experimental estimation, considering 5°C as a reference and the quantification of the change up to 10°C.

**Fig. 5:**
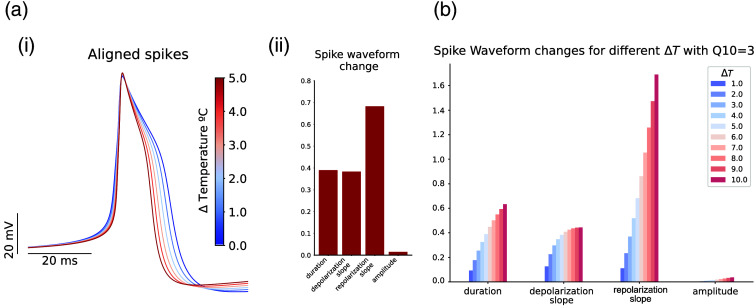
Waveform change in the CGC-model due to ΔT temperature variation. (ai) The spike waveform superposition for distinct ΔT values. Spikes are aligned to the initial value of each waveform. (aii) The normalized change in the waveform is depicted for all metrics (duration, depolarization, and repolarization slopes and amplitude). (b) The change in response to temperature variation from 1°C to 10°C ($Q_{10}=3$) in the normalized metrics.

Figure 5 shows the change in the spike waveform caused by variations in temperature. The $Q_{10}$ factor was added to every dynamical equation in the model (i.e., conductances, activation gates, and capacitance, see Sec. 2.8.1). In Fig. 5(a), we show the changes in the waveform for ΔT=0°C to 5°C, represented as superimposed waveforms in (ai), and its quantification in (aii) normalized to the maximum, which is analogous to the previous sections (see Figs. 4 and 2): |max−min|/|max|. Note how both the spike waveform shape and the quantification of the changes are similar to the experimental results. We can observe changes in duration, depolarization, and repolarization slopes, with a very small change in amplitude. The modulation obtained by combining these parameters was not achieved by tuning them separately. It is important to highlight that as the temperature increased (red lines), the spike got narrower by the corresponding alteration in slopes and duration, which supports the hypothesis that the observed effect in single neurons of *Lymnaea stagnalis* might be, to a large extent, caused by temperature gradient. In Fig. 5(b), there is a comparison of different temperature changes for the same $Q_{10}$ value in the model. Note that the relation of each parameter to the change in temperature is different, being the repolarization slope the one with the strongest relation, increasing much more rapidly than the duration or the repolarization slope. This points to a main role in the change from some of the channels, especially those that have more tolerance to change. This relation in the repolarization is similar to the comparison of the two neuron types analyzed in the experimental results [Fig. 2(a)], where the main difference was present in the repolarization.

Analogously to the simulations in the previous sections, we characterized the variations in the waveforms for each individual candidate, varying the temperature only for one ionic channel at a time, with a similar result as in Fig. 4, where no candidate alone could reproduce the effect (see Fig. S2 in the Supplementary Material). To further explore these candidates and relations, we repeated the model simulation with temperature variations up to 5°C but canceling the temperature dependency in one channel at a time. Note that overriding the temperature dependence of some of the channels at a time is only possible in a theoretical environment like this, which allows us to expose the role of each channel in relation to the temperature modulation. The results are also reported in (Fig. S2 in the Supplementary Material) showing the waveform variations for temperature dependency for one channel at a time, and its suppression for one channel at a time, along with the quantification of the change as the percentage of change (see Sec. 2.7).

Exploring the waveform change during temperature variation showed that the most similar change to the experimental laser modulation was reached in the models with the temperature dependency description in all parameters. We showed that, for most currents as the temperature increases, the experimentally observed modulation relations were maintained, changing in duration and repolarization slope, with a larger change in repolarization slope and a mild change in amplitude. This is also supported by excluding temperature dependency from isolated ion channels and proposing temperature dependency for one channel at a time (Fig. S2 in the Supplementary Material). Furthermore, it is in agreement with the results of the modeling sweeping the parameter space for distinct candidates without a specific description of the temperature (see Sec. 3.2), which showed that no modulation of an individual candidate but a combination of them can explain the experimental quantification.

### 3.3 Activity-Dependent Stimulation to Assess the Laser Effect at Distinct Stages of the Spike Dynamics

So far we have shown how sustained CW-NIR laser affects neural activity by modifying the dynamics of spike generation. In Sec. 3.2, we used a conductance-based model to theoretically assess the spike evolution and the different candidates involved in the modulation of the AP generation (i.e., ionic channels and capacitance). To address this effect in an experimental setting is a complex task. Usually, it is accomplished using chemicals to block or open specific channels.^13^ This is not a generalizable method in different systems and individuals, and it restricts the channel study to a system with a detailed description of the specific neuron being recorded. We chose instead to assess the spike generation dynamics at different stages, which implies modifying the activity of several channels at a time in a precise timing relative to the spike generation dynamics. This task is only experimentally feasible with an activity-dependent stimulation protocol.

In infrared stimulation literature, the most spread technique has been pulsed illumination which stimulates at a fixed frequency. Although this approach has been effective in some tasks, such as eliciting neural activity, it has limited possibilities in the context of precision and adaptability. Thus, with the activity-dependent protocol proposed here, we also provide an open-access alternative to the widely used fixed-frequency pulsed laser stimulation protocols, which usually depend on a specific combination of restrictions from manufacturers, controllers, and diode laser availability. In addition, a closed-loop approach provides further means to deal with the history-dependent nature of neural dynamics and its partial observability.^34^

Here, we propose a closed-loop stimulation protocol where we can differentiate between the phases of the AP and illuminate the neurons only at certain intervals of the spike generation dynamics. In this protocol, the laser illumination was controlled by a mechanical shutter triggered by the prediction of events in the voltage signal. A real-time software system ran the prediction algorithm and triggered the illumination for short periods of time at different phases of the spike generation when distinct channels were active [see Sec. 2.9 and Fig. 1(e)]. The prediction of the events was computed by two algorithms, one based on a voltage threshold updated at each spike peak occurrence and a second one that calculated the voltage area from the hyperpolarization (minimum) to the next hyperpolarization. Based on this prediction, the illumination was triggered at the specified time before the spike occurrence (see Sec. 2.9 for details on these algorithms). The implementation of these algorithms is available as a module for the real-time, open-source system RTXI^43^ in a GitHub repository available at: https://github.com/GNB-UAM/spike_predictor/.

**Fig. 6:**
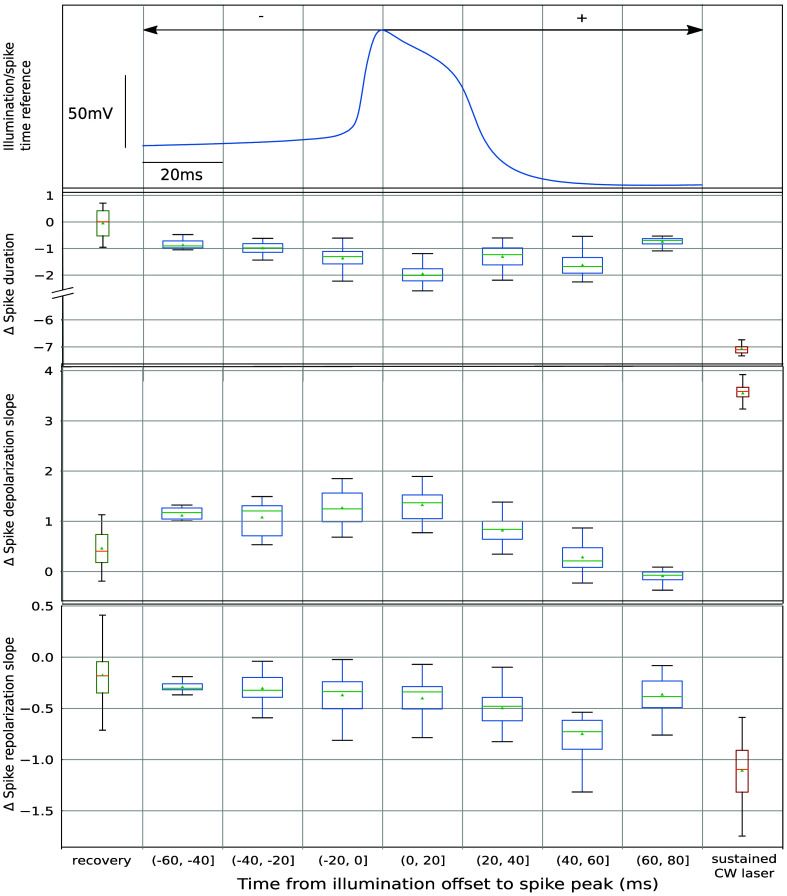
Study of the laser effect at different stages of the spike waveform with an activity-dependent stimulation protocol. The panels quantify the change induced by the laser stimulation at distinct illumination offsets, time intervals from the end of the illumination to the peak of the spike. Top panel shows a spike waveform from the experiment as a time reference for the offset—time 0 corresponds to the spike peak. Boxplots represent the difference of each metric with respect to the control. All illumination intervals, pictured in the blue boxes, had the same duration of 58 ms, and spikes were grouped by the illumination offset. Recovery and continuous laser reference are also shown in green and red boxes at the left and right in the figure, respectively. The spike metrics selected here were duration, depolarization, and repolarization slopes, second, third, and fourth rows, respectively.

Figure 6 shows the outcome of the application of this closed-loop protocol, with a stimulation interval lasting 58 ms. The timeline in the figure represents the offset of the illumination, i.e., the time that corresponds to the end of an illumination interval to the peak of the AP [see an illustration exemplifying the illumination offset in Fig. 1(f)]. The offsets were in the range from 60 ms before the AP peak up to 80 ms after its occurrence (this wide range is required because of the natural slow dynamics of *Lymnaea stagnalis*’s neurons). Each row in the figure represents the change in relation to the mean of the respective control trials for every illumination range. The change is represented for the three metrics in which we observed modulation during sustained illumination—duration, repolarization, and depolarization slopes, respectively. The different stimulation intervals are grouped by the time offset from the illumination to the peak of the AP. The spike shown in the figure is plotted as a reference of the phase of the AP in which the illumination finished. Recovery and sustained laser references are also represented at the left and right of each row, in green and red, respectively. For the three metrics here displayed, we can see how as the illumination offset got closer to the spike, the change was larger, and then recovered, as the illumination interval covered less the AP, resulting in an arch-shaped trend. Although this trend is visible for the three parameters characterized, it is manifested to a different degree in each of them. Note also that there was a temporal shift of the laser effect depending on the instant of stimulation. The maximum change value and the initialization of the recovery were different for the depolarization and for the repolarization. The effect on each of these metrics directly depended on the spike phase when the laser was illuminating the neuron. Thus, this variation of the laser effect points to a distinct modulation on each channel. The magnitude of the change under the sustained laser stimulation was larger than that observed at any of the phases addressed with the activity-dependent protocol. This may be caused by a heating delay during the stimulation, although there was no difference between the first and last spikes in the sustained laser, the opening time of the laser shutter might have been smaller than the heating time necessary for the neuron to reach the maximum effect.

**Fig. 7:**
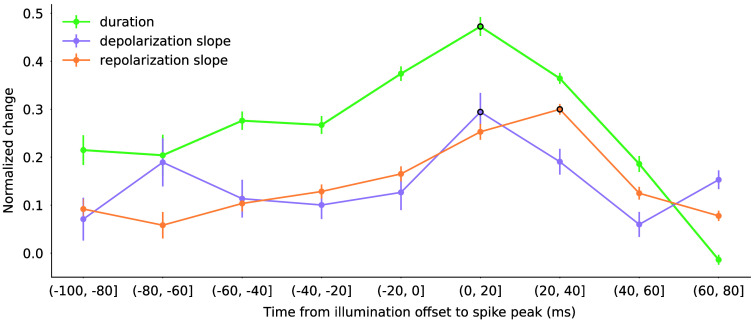
Normalized change for grouped values of spike duration, depolarization, and repolarization slopes at distinct illumination offsets in the activity-dependent stimulation protocol. Each value in each group was normalized to the mean of its corresponding day controls as a minimum value and the mean of the continuous laser recordings for each day as the maximum value. The maximum value for each metric is marked by a black circle.

Figure 7 agglutinates the results from five different closed-loop experiments, all of them normalized to the mean of the control and sustained laser references for each day, as minimum and maximum values, respectively. The arch trend is maintained. Again, note that the maximum effect for each spike metric occurs at a different stage. For the depolarization, the maximum change was found at the range of −20 to 0 ms, which corresponds to stimulation during the whole depolarization. A fast rise when the illumination ceased right after the spike can be seen (i.e., (0–20] range), since it corresponded to stimulation during the depolarization and repolarization. In the repolarization, this trend was slightly delayed, reaching the maximum difference from the control at (20–40]. Such changes were also reproduced in the duration. This points to a modulatory effect of the laser depending on the stimulation instant. Previous to −20  ms, the laser was illuminating the neuron while all ionic channels were starting to activate, especially those involved in the process of depolarization. However, those channels involved in the repolarization and hyperpolarization were also active earlier than the peak. This is why we can see a difference in all three metrics even at the early ranges of the AP generation (e.g., −80 ms).

Using the activity-dependent protocol, we were able to assess the neural activity at different stages of its dynamics in a controlled way. The results from these experiments showed that it is possible to modify the AP generation in a temporally precise manner and that the effect of the CW-NIR laser illumination is dependent on the instant of the stimulation. This sets the basis for assessing the biophysical sources of the effect impacting distinct channels without modifying the system condition. Also, it is a proof of concept demonstrating the possibility of developing laser stimulation protocols driven by specific neural activity events in an accessible and freely available real-time tool.

## 4 Discussion

### 4.1 Singularity of the Sustained and Activity-Dependent CW-NIR Stimulation on Neural Dynamics

Advantages of infrared laser neuromodulation beyond its non-invasive nature include its relative simplicity regarding stimulation protocol design, good penetration depth, and the possibility to implement highly selective spatio-temporal stimulation delivery. The effectiveness of future applications will depend on a clear understanding of the mechanisms of the neural dynamics modulation.

Most previous studies used protocols involving high-frequency pulsed lasers under the assumption that CW laser stimulation paradigms do not provide significant activation or neuromodulation.^11^^,^^14^^,^^18^^,^^24^^,^^63^^,^^64^ Their focus involved heating the neurons to elicit spiking activity. The laser wavelengths used were mostly in the range of 1800 nm, close to a water absorption band. Here, we explored a different approach, using a 830 nm CW-NIR laser in sustained and activity-dependent triggered stimulation instead of pulsed illumination at a fixed frequency. This setup has a promising future for clinical applications for long-term stimulation and patient-based treatments.

We assessed the action of sustained and activity-dependent CW-NIR stimulation to unveil the biophysical sources of the observed modulation of neuronal dynamics. We combined experimental and theoretical methods to analyze this effect. First, we quantified the change in AP waveform dynamics and on the inter-spike intervals by comparing triplets of long intracellular recordings of control, laser stimulation, and recovery. We found that sustained exposure to 830 nm CW laser effectively modulated the spike waveform in a reversible manner. We showed this modulation in two different neuron types, illustrating the generalization of the effect. We observed a stronger effect on duration and repolarization, followed by a less strong change in the depolarization slope and a minimal change in amplitude. The neuron dynamics were restored after stimulation. It is important to highlight that here we presented modulation of tonic spontaneous activity, not elicitation of spiking activity as in most previous studies.^11^^,^^12^^,^^17^^,^^45^ We also showed a tendency to increase the spiking activity under sustained stimulation, not limited to a specific time/intensity configuration of laser pulses as it is most frequently done in the literature.^12^^,^^14^^,^^24^^,^^65^ Although there are previous studies discussing the inhibitory ability of infrared-laser illumination,^19^^,^^66^^–^^68^ we did not find evidence of any direct CW-NIR inhibitory effect. Note that the origin of tonic spiking was affected by the intrinsic properties of the cell and the synaptic inputs within the circuit, e.g., the illuminated neuron might be triggering inhibitory or excitatory feedback from other neurons, complicating the analysis. This explains the lack of excitation in a subpopulation in Fig. 3. Spontaneous neural activity and the nature of the living preparation used may naturally tend to decrease the firing rate.

### 4.2 Biophysical Explanation of the CW-NIR Modulation Through Modeling and Activity-Dependent Stimulation

The results of sustained CW-NIR illumination alone cannot discard previously suggested mechanisms, such as cytochrome oxidase^22^^,^^23^ or calcium release from internal storage.^29^ The fact that the illumination directly affects the spike waveform but does not always translate into an increased firing rate may indicate that there is more than a single mechanism involved. Moreover, our analysis of sustained laser stimulation does not point to a slow change such as the one expected with the liberation of ${\mathrm{Ca}}^{2+}$ caused by a mitochondrial modulation,^28^^,^^29^ since we observed a minimal delay between the illumination onset and the modulatory effect, and the illumination cessation and the recovery. The short exposure in the activity-dependent experiment with quick response time also points to a short timescale effect, such as a direct effect on the ionic channels.

Conductance-based models allowed us to identify the most compatible biophysical explanation to the CW-NIR modulation. We evaluated the capacitance and distinct ionic channels in the parameter space of three conductance-based models, which would be highly costly experimentally. We concluded that all candidates explored contributed to partial reproduction of the waveform modulation, but none was sufficient to explain the full observed effect. Capacitance is one of the most discussed candidates.^17^^,^^18^^,^^26^^,^^69^ However, in our modeling study, capacitance alone was not able to reproduce the modulation. Although the isolated modification of any channel resulted in a limited explanation of the CW-NIR change, late activation channels such as potassium—preserving the depolarization-repolarization change relation—or high-activated calcium—necessary for shoulder-shaped modulation—seem to play a key role in reproducing the observed effect.

Temperature-dependent simulations validated that the best explanation for the sustained laser action is a combined modulation of channels, reproducing the observed change for amplitude, duration, and slopes. This supports previous studies’ hypothesis that the photo-thermal interaction is key in the NIR laser effect.^15^^,^^16^^,^^25^^,^^27^^,^^44^^,^^45^^,^^53^ We selectively excluded one channel from the temperature dependency at a time in the CGC-model, which cannot be performed experimentally. We found $I_{D}$ and $I_{\mathrm{NaP}}$ channels to be critical for the activity modulation, radically changing the waveform when altering temperature dependency. Also, canceling the temperature dependency of $I_{\mathrm{HVA}}$ largely changed the amplitude, indicating its importance in preserving the observed amplitude-repolarization relation during the modulation.

Finally, a closed-loop protocol allowed altering the AP at distinct generation phases. We presented a new open-source protocol for spike prediction to stimulate at precise times around the occurrence of the APs. The outcome of the CW-NIR effect at distinct time intervals in relation to the timing of the spike’s peak highlighted the importance of the stimulus delivery time. By changing the illumination instant, we shifted the effect on the waveform shape, getting different maximum metric changes at different stages of the AP generation.

These changes in the waveform open a discussion about the biophysical source of this effect. With short closed-loop illumination intervals (<60  ms), we observed a controlled modulation of neural activity smaller than the effect during the sustained laser illumination. In the open-pipette estimation, the maximum change for the steady-state temperature value (1°C to 2°C) was reached after 1 s and the change after 50 ms was only of 0.1°C. This estimation was performed on the preparation’s solution, and our laser wavelength is far from the intense water absorption bands. So temperature change could be higher in the neuronal membrane, as the specific heat capacity of the water is ∼30% larger than the estimated on the membrane.^62^ This would result in a faster temperature increase under heat-inducing stimulation. Also, in the model, we observed a change similar to the experimental results with ΔT≥5°C. Thus, the modulation might not be caused by simply heating the surrounding water. In the sustained stimulation, there might be additional modulation sources such as an effect on the mitochondria as it has been discussed in previous studies,^23^^,^^28^^,^^29^ which we cannot discard as adding to the modulation of ionic channels. However, the effect observed during the activity-dependent stimulation is unlikely to have other than fast sources, such as ionic-channels. A rigorous characterization of the timescale of the temperature changes induced by the CW-NIR and the associated instantaneous voltage dynamics could provide further insight on the fast and slow biophysical mechanisms underlying the waveform modulation. This is particularly relevant for the design of fast activity-dependent protocols to produce the observed effect safely with minimal biophysical perturbation. An accurate characterization of the relation between temperature and neuronal dynamics under CW-NIR stimulation requires novel highly precise protocols to measure the membrane temperature and fast non-periodic electro-optical shutters controlled by real-time software technology.

### 4.3 Applications for Research and Clinical Use

The open-source approach described in this paper can be generalized for any animal and preparation. In addition, our protocol leaves plenty of possibilities for other closed-loop stimulation methodologies, including clinical interventions. We provided an open-access repository with the code to reuse our protocols and the module for RTXI, which can be used with any control hardware including fast electro-optical shutters.

Regarding the non-invasive nature of the CW-NIR laser effect, we could not observe any damage to the cells linked to the stimulation in our experiments. We can hypothesize that stimulation from a laser with higher power could be tolerated by neurons. The recovery of the neural dynamics after illumination does not mean that the CW-NIR laser stimulation cannot be employed to address laser-driven plasticity in protocols designed for this goal. We have shown that sustained CW-NIR laser effectively accelerates neural dynamics in single neurons affecting a combination of biophysical mechanisms. Also, our results indicate that novel research and clinical applications of the excitability increase of laser stimulation must rely on a careful selection of the stimulus parameters and the timing of the illumination. In this context, the results of our pioneer activity-dependent infrared laser stimulation provide a novel approach to adapt the modulation of neural dynamics to specific applications, particularly in the field of personalized treatments including stimulation-driven plasticity.

## Supplementary Material



## Data Availability

Experimental data, analysis tools, and closed-loop software used for this study are available at the following links: github.com/GNB-UAM/Garrido-Pena_Modulation-neural-dynamics-by-CW-NIR-stimulation RTXI module Spike_predictor (github.com/GNB-UAM/spike_predictor).
