# Supplementary material for: Modulation of neuronal dynamics by sustained and activity-dependent continuous-wave near-infrared laser stimulation
Source: Neurophotonics. 2024 May 17;11(2):024308. doi: 10.1117/1.NPh.11.2.024308 (PMC11100521; doi:10.1117/1.NPh.11.2.024308)
Supplement: Supplementary file 1 [file NPh_011_024308_SD001.pdf]

Modulation of neuronal dynamics by sustained and activity-dependent continuous-wave near-infrared laser stimulation

Alicia Garrido-Peña, Pablo Sanchez-Martin, Manuel Reyes-Sanchez, Rafael Levi, Francisco B. Rodriguez, Javier Castilla, Jesus Tornero, Pablo Varona.

Neurophotonics 2024

## Supplemental Figure S1.

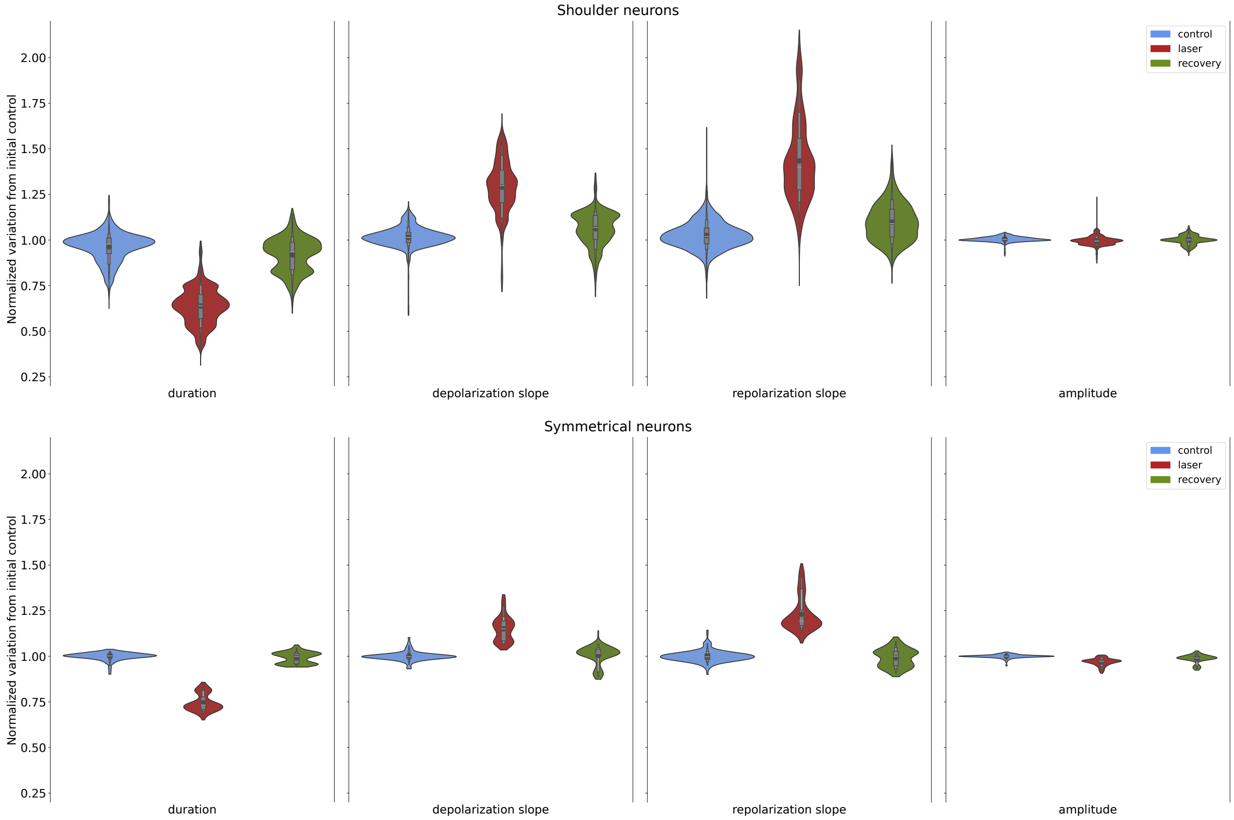


**Supplemental Figure 1: Segregation of data into shoulder and symmetrical neural types**. Violin plots for the four metrics -duration, depolarization slope, repolarization slope and amplitude- grouped by control, laser and recovery trials. Spikes metrics are normalized to the first control, as in Figure 2 panel C.

**Supplemental Figure S2.**

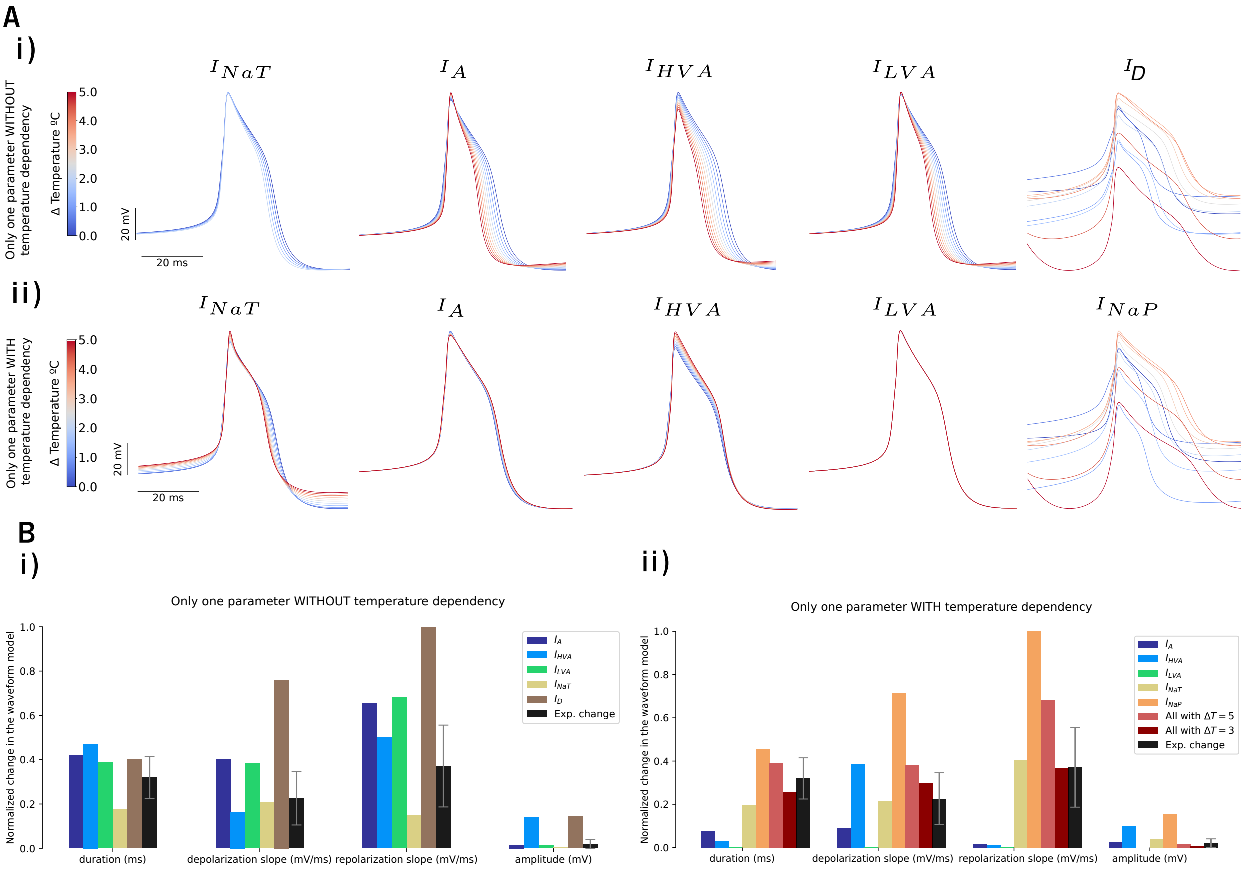


**Supplemental Figure 2: Simulation of the effect of individual channels on the spike waveform under temperature modulation.** Panel A.i) Waveform modulation in the CGC-model excluding temperature dependency in one channel at a time, from left to right: $I_{\text{NaT}}$, $I_A$, $I_{\text{HVA}}$, $I_{\text{LVA}}$, $I_D$ respectively. Panel A.ii) Waveform modulation in the CGC-model with temperature dependency only in one channel at a time, from left to right: $I_{\text{NaT}}$, $I_A$, $I_{\text{HVA}}$, $I_{\text{LVA}}$ and $I_{\text{NaP}}$, respectively. Note that $I_{\text{NaP}}$ is not in panel A.i) and neither is $I_D$ in A.ii), as for these two particular simulations there was no spike generation. Panels B.i) and B.ii) show the quantification of the waveform change for $\Delta T = 5°C$ in duration, depolarization slope, repolarization slope and amplitude for all channels in panel A. Both figures include, as reference, the experimental mean and STD (showed in black)

and, for case B.ii) (only one channel at a time), the quantification when all channels have the same temperature dependency (all with $\Delta T = 5°C/3°C$, data used in Figure 5) is depicted in light and dark red bars. Note that no candidate alone could reproduce the observed experimental laser modulation but some channels have a more direct relation to the temperature change such as $I_{\text{LVA}}$ for the repolarization or $I_D$ channel, crucial for the spike generation waveform.
